# Supplementary material for: Cadmium-Inspired Self-Polymerization of {LnIIICd2} Units: Structure, Magnetic and Photoluminescent Properties of Novel Trimethylacetate 1D-Polymers (Ln = Sm, Eu, Tb, Dy, Ho, Er, Yb)
Source: Molecules. 2021 Jul 15;26(14):4296. doi: 10.3390/molecules26144296 (PMC8307922; doi:10.3390/molecules26144296)
Supplement: Supplementary file 1 [file molecules-26-04296-s001.zip › molecules-1224942-supplementary.pdf]

Supplementary materials

Cadmium-Inspired Self-Polymerization of $\{Ln^{III}Cd^{II}_2\}$ Units: Structure, Magnetic and Photoluminescent Properties of Novel Trimethylacetate 1D-Polymers (Ln = Sm, Eu, Tb, Dy, Ho, Er, Yb)

Maxim A. Shmelev[1], Ruslan A. Polunin [2,¥] Natalia V. Gogoleva [1], Igor S. Evstifeev [1], Pavel N. Vasilyev[1], Alexey A. Dmitriev [3], Evgenia A.Varaksina [4], Nikolay N. Efimov [1], Ilya V. Taydakov [4,5], Alexey A. Sidorov[1], Mikhail A. Kiskin [1,*], Nina P. Gritsan [3], Sergey V. Kolotilov[2], Igor L. Eremenko [1]

[1] N. S. Kurnakov Institute of General and Inorganic Chemistry, Russian Academy of Sciences, 31 Leninsky prosp., 119991 Moscow, Russia
[2] L. V. Pisarzhevskii Institute of Physical Chemistry of the National Academy of Sciences of Ukraine, 31 prosp. Nauki, 03028 Kiev, Ukraine
[3] V. V. Voevodsky Institute of Chemical Kinetics and Combustion, 3 Institutskaya Str., 630090 Novosibirsk, Russia
[4] P. N. Lebedev Physical Institute, Russian Academy of Sciences, 53 Leninsky prosp., 119991 Moscow, Russia
[5] Academic Department of Innovational Materials and Technologies Chemistry, Plekhanov Russian University of Economics, 117997 Moscow, Russia
[¥] Deceased.
* Correspondence: mkiskin@igic.ras.ru

I. Structural data
II. PXRD data
III. Magnetic data

I. Structural data

Table S1. Selected crystal data and parameters for structure refinement of the compounds **1-4, 6** and **7**.

	**1**	**2**	**3**	**4**	**6**	**7**
Empirical formula	$C_{37}H_{70}Cd_2NO_{16}Sm$	$C_{37}H_{70}Cd_2NO_{16}Eu$	$C_{37}H_{70}Cd_2NO_{16}Tb$	$C_{37}H_{70}Cd_2NO_{16}Dy$	$C_{37}H_{70}Cd_2NO_{16}Er$	$C_{37}H_{70}Cd_2NO_{16}Yb$
Formula weight	1160.09	1161.70	1168.66	1172.24	1177.00	1182.78
T (K)	150(2)	150(2)	296(2)	120(2)	296(2)	150(2)
Crystal system	Monoclinic					
Space group	$P2_1/n$					
a (Å)	11.2163(10)	11.1940(5)	11.1951(3)	11.1541(4)	11.1758(12)	11.1592(4)
b (Å)	20.3091(18)	20.2542(10)	20.2177(6)	20.1505(7)	20.114(2)	20.0749(5)
c (Å)	22.7499(19)	22.7372(11)	22.7620(6)	22.7275(8)	22.793(2)	22.7695(7)
β (º)	101.995(2)	101.8694(7)	101.8610(10)	101.8983(7)	101.863(2)	101.850(1)
V(Å^3)	5069.1(8)	5044.9(4)	5041.9(2)	4998.5(3)	5014.1(9)	4992.1(3)
Z	4					
D_{calc} (g·cm^{-3})	1.520	1.530	1.540	1.558	1.559	1.574
μ (mm^{-1})	2.033	2.122	2.282	2.382	2.558	2.762
θ range (º)	1.36–28.30	1.36–28.33	1.36–28.25	1.83–27.88	2.03–29.57	2.22–29.57

Range of *h*, *k* and *l*	−14→14<br>−27→27<br>−30→22	−14→14<br>−26→26<br>−29→30	−14→13<br>−26→26<br>−29→30	−14→14<br>−26→26<br>−29→29	−15→15<br>−27→25<br>−31→30	−13→12<br>−24→24<br>−28→25
T_{min}/T_{max}	0.591/0.698	0.612/0.746	0.641/0.746	0.516/0.635	0.591/0.738	0.607/0.746
F(000)	2340	2344	2352	2356	2364	2372
Number of parameters	614	530	610	596	554	567
Reflections collected	40736	51468	37961	92663	41449	39740
Unique reflections	12482	12509	12399	11923	13288	9808
Reflections with $I > 2\sigma(I)$	9695	10119	10401	10211	9547	6973
R_{int}	0.0441	0.0502	0.0305	0.0437	0.0557	0.0798
GooF	0.975	1.077	1.198	1.045	0.937	1.013
$R_1(I > 2\sigma(I))$	0.0350	0.0407	0.0267	0.0230	0.0394	0.0454
$wR_2\,(I > 2\sigma(I))$	0.0784	0.1104	0.0616	0.0500	0.0825	0.0768

Table S2. Continuous Shape Measures (CShM) values for the potential coordination polyhedra of Ln in the structure of complexes **1**-**4**, **6** and **7**.

	**1** ($SmCd_2$)	**2** ($EuCd_2$)	**3** ($TbCd_2$)	**4** ($DyCd_2$)	**6** ($ErCd_2$)	**7** ($YbCd_2$)
Octagon, D_{8h}	28.865	29.088	29.132	29.278	29.420	29.490
Heptagonal pyramid, C_{7v}	21.499	21.695	21.790	21.871	22.086	22.261
Hexagonal bipyramid, D_{6h}	12.352	12.547	12.601	12.751	12.912	13.136
Cube, O_h	13.498	13.626	13.560	13.674	13.690	13.678
Square antiprism, D_{4d}	4.767	4.659	4.604	4.526	4.456	4.339
Triangular dodecahedron, D_{2d}	3.315	3.157	3.028	2.941	2.791	2.633
Johnson gyrobifastigium J26, D_{2d}	11.209	11.197	11.101	11.057	11.083	11.109
Johnson elongated triangular bipyramid J14, D_{3h}	25.360	25.448	25.539	25.598	25.765	25.942
Biaugmented trigonal prism J50, C_{2v}	3.837	3.693	3.617	3.508	3.414	3.296
Biaugmented trigonal prism, C_{2v}	3.274	3.148	3.101	3.019	2.971	2.868
Snub diphenoid J84, D_{2d}	4.204	4.065	3.896	3.801	3.660	3.517
Triakis tetrahedron, T_d	14.283	14.421	14.356	14.322	14.301	14.274

Elongated trigonal bipyramid, D_{3h}	21.461	21.643	21.885	22.097	22.325	22.557

II. PXRD data

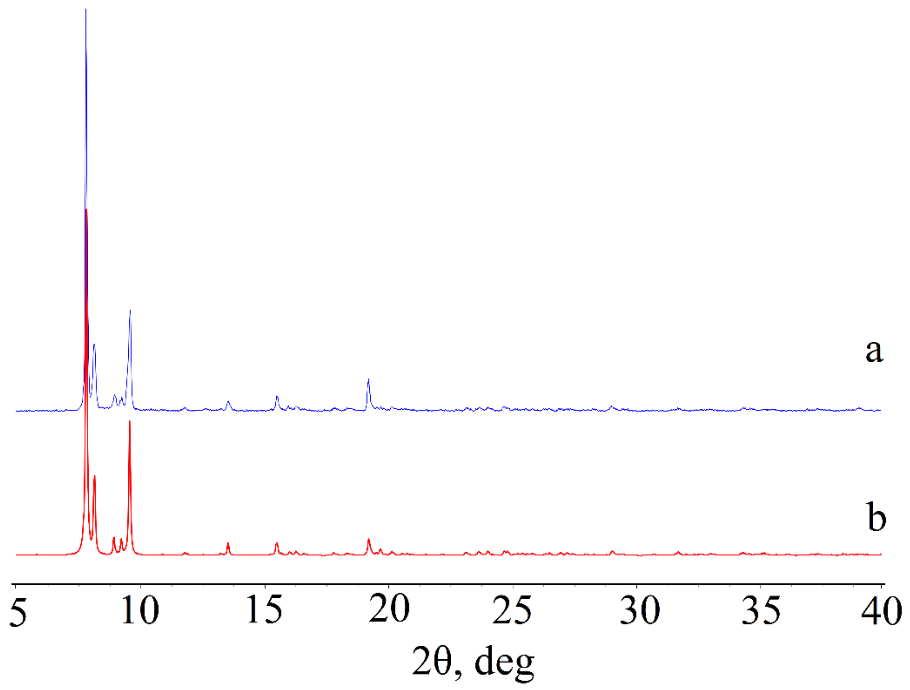

Figure S1. Experimental (a) and calculated (b) diffractograms for compound **1**.

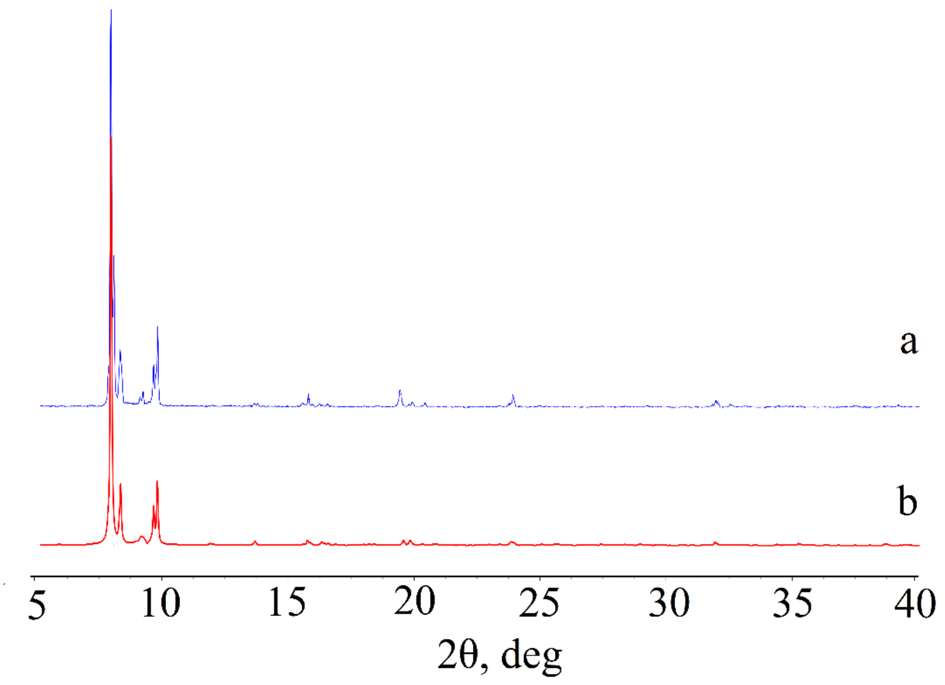

Figure S2. Experimental (a) and calculated (b) diffractograms for compound **2.**

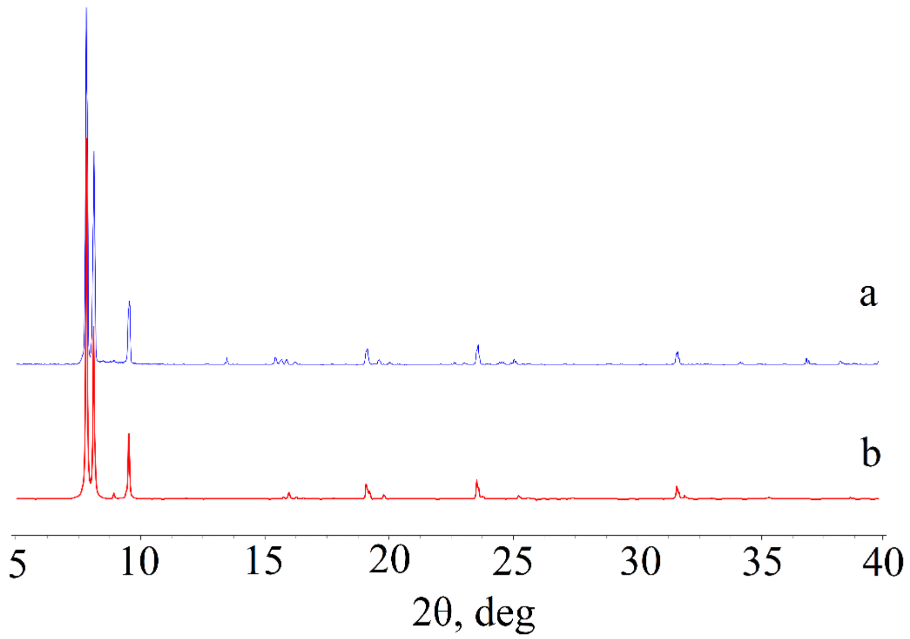

Figure S3. Experimental (a) and calculated (b) diffractograms for compound **3.**

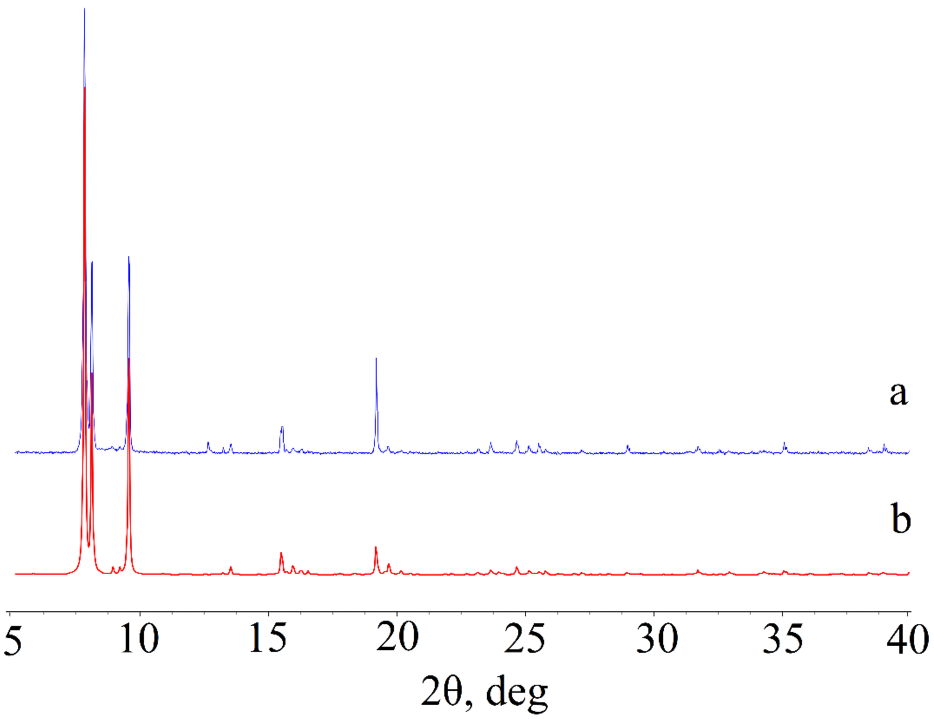

Figure S4. Experimental (a) and calculated (b) diffractograms for compound **4.**

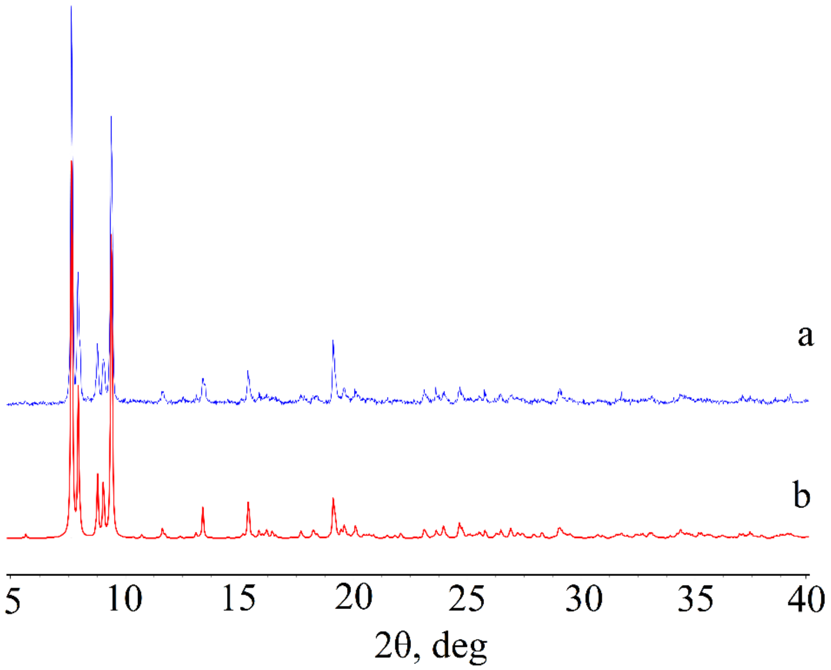

Figure S5. Experimental diffractogram for compound **5** and calculated diffractogram for compound **6** (b).

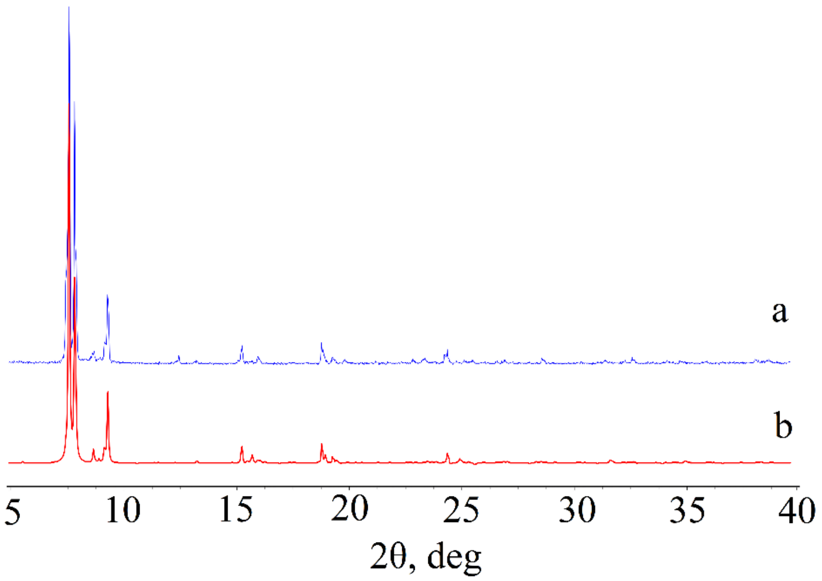

Figure S6. Experimental (a) and calculated (b) diffractograms for compound **6.**

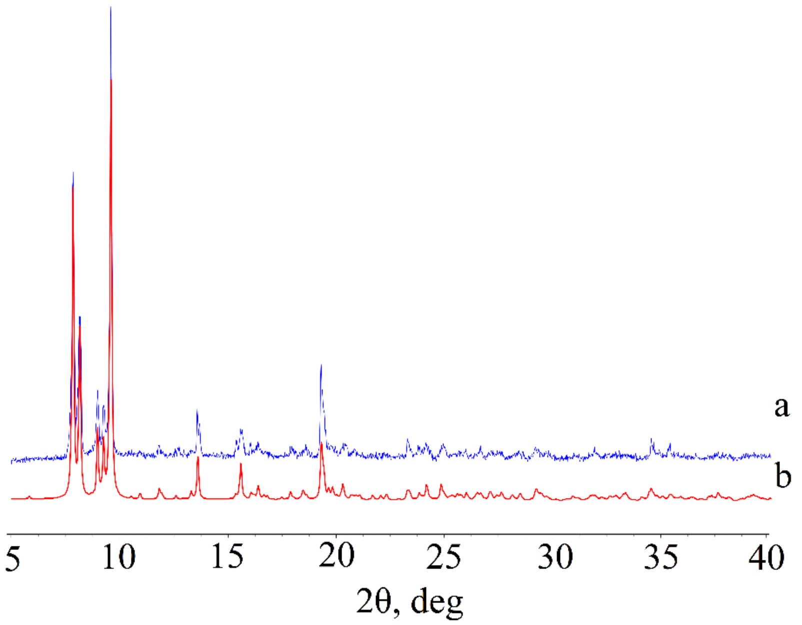

Figure S7. Experimental (a) and calculated (b) diffractograms for compound **7.**

III. Magnetic data

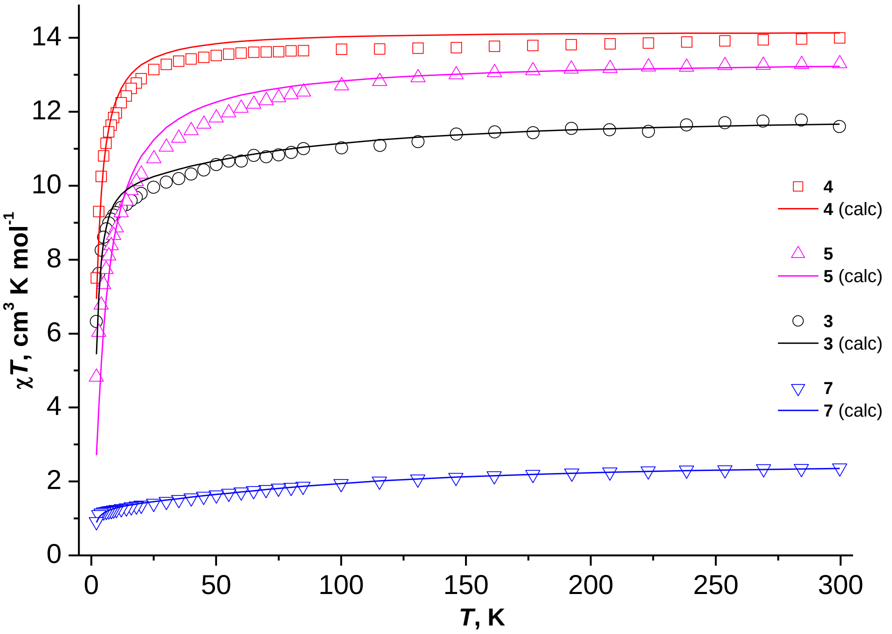

Figure S8. Temperature dependence of $\chi_M T$ for **3**–**5** and **7** (H = 5000 Oe). The solid lines are the results of simulations using the Hamiltonian (1) (see main text).

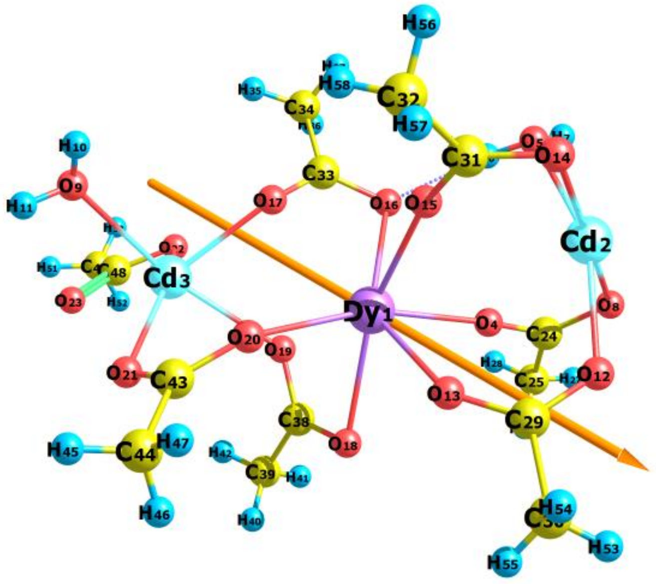

Figure S9. Molecular structure of the model $DyCd_2$ cluster (**4m**) used in the calculations and the direction of the *easy* axis in the ground Kramers doublet, evaluated using the SINGL_ANISO code and the results of SA-CASSCF(9,7)/SO-RASSI/ANO-RCC-VTZP calculations.

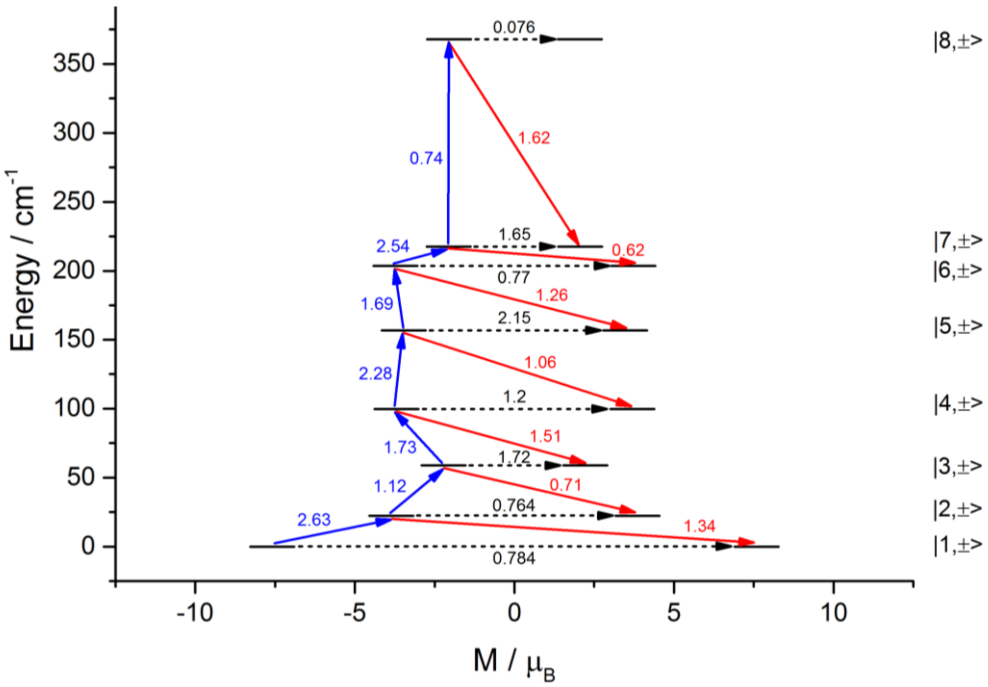

Figure S10. Magnetization blocking barrier in cluster Cd_2Dy calculated at the SA-CASSCF(9,7)/SO-RASSI/ANO-RCC-VTZP level using the SINGLE-ANISO code. The states are arranged according to the values of their magnetic moments. The arrows show the connected exchange states, and the number at each of them stands for the corresponding matrix element of the transversal magnetic moment.

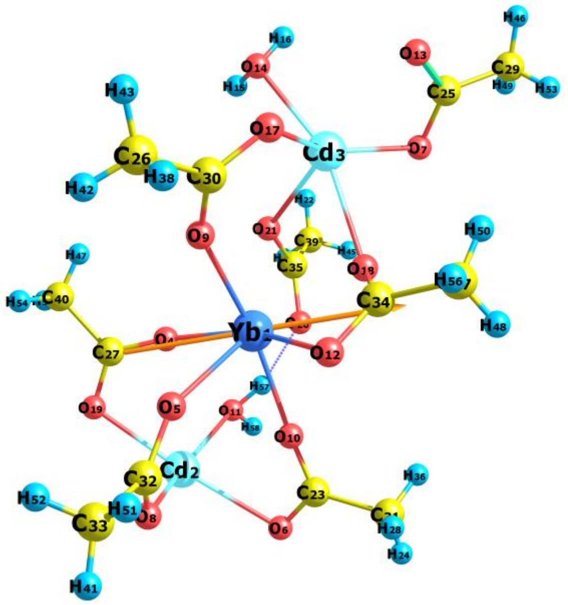

Figure S11. Molecular structure of the model $YbCd_2$ cluster (**7m**) used in the calculations and the direction of the *easy axis* in the ground Kramers doublet, evaluated using the SINGL_ANISO code and the results of SA-CASSCF(13,7)/SO-RASSI/ANO-RCC-VTZP calculations.

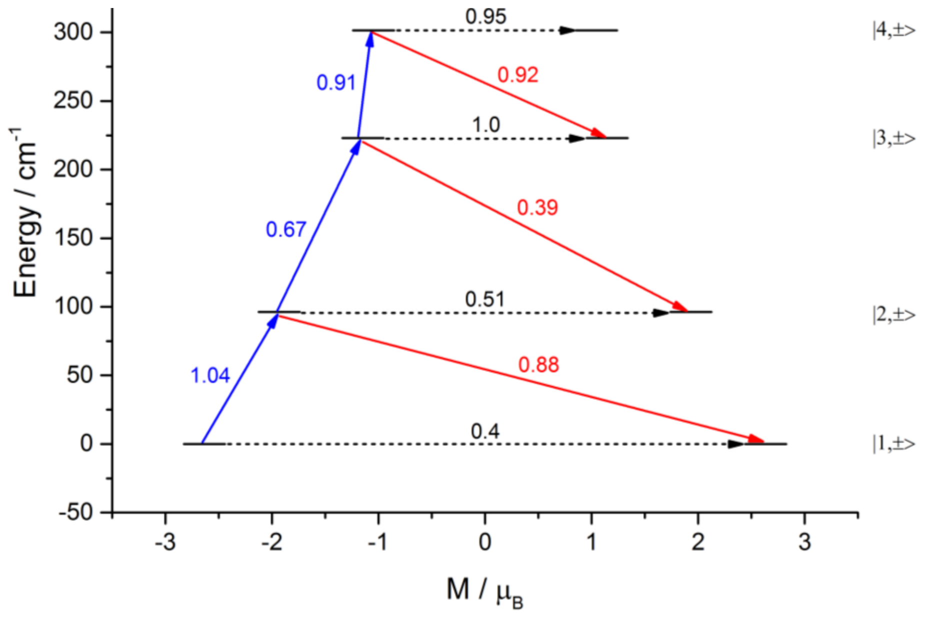

Fig. S12. Magnetization blocking barrier in cluster Cd_2Yb calculated at the SA-CASSCF(13,7)/SO-RASSI/ANO-RCC-VTZP level using the SINGLE-ANISO code. The states are arranged according to the values of their magnetic moments. The arrows show the connected exchange states, and the number at each of them stands for the corresponding matrix element of the transversal magnetic moment.

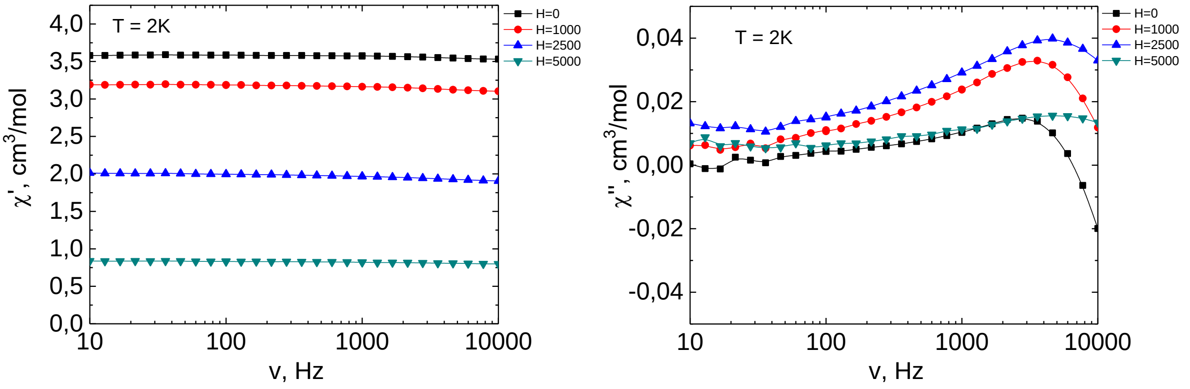

Figure S13. Frequency dependences of the real χ' (left) and imaginary χ'' (right) components of the magnetic susceptibility of complex **3** in various applied fields. Solid lines are visual guides.

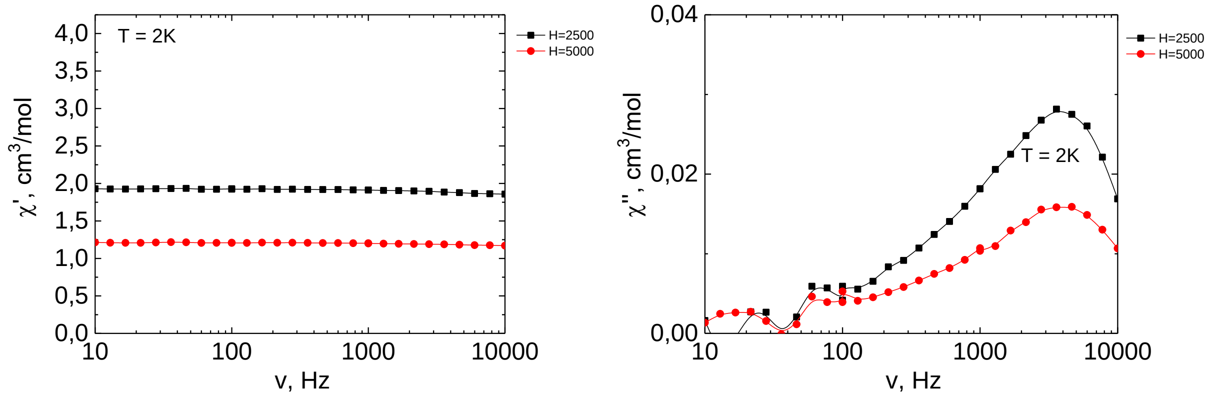

Figure S14. Frequency dependences of the real χ' (left) and imaginary χ'' (right) components of the magnetic susceptibility of complex **5** in various applied fields. Solid lines are visual guides.
